# A scavenging double mask to reduce workplace contamination during mask induction of inhalation anesthesia in dogs

**DOI:** 10.1186/1751-0147-53-1

**Published:** 2011-01-13

**Authors:** Susanne Friembichler, Paul Coppens, Heli Säre, Yves Moens

**Affiliations:** 1 Division of Anesthesiology and Perioperative Intensive Care, University of Veterinary Medicine, Vienna, Austria

## Abstract

**Background:**

Workplace contamination by the use of volatile anesthetic agents should be kept to a minimum if a potential health hazard is to be minimised. Mask induction of animals is a common procedure. The present study investigates the efficiency of a novel scavenging double mask in reducing waste gas concentrations in the breathing zone of the anesthetist performing this procedure.

**Methods:**

Twelve beagle dogs (ASA I) undergoing general anesthesia for a dental procedure were intravenously premedicated with medetomidine and butorphanol (10 μg/kg and 0.2 mg/kg). Anesthesia was induced via a custom-made scavenging mask using isoflurane in oxygen. In six dogs (group S), scavenging from the mask was performed whereas in six other dogs (group NS) the scavenging function was disabled. Isoflurane concentration was continuously measured with photoacoustic spectroscopy at the level of the shoulder of the anesthetist before and during mask induction and additionally during intubation. Statistical analysis was performed with a Student t- test and a Mann-Whitney U test (p < 0.05 for significance).

**Results:**

The mean isoflurane concentration during baseline (premedication) was 1.8 ± 0.8 ppm and 2.3 ± 0.6 ppm in group S and NS respectively. This increased during mask induction to 2.0 ± 0.8 ppm and 11.2 ± 6.0 ppm respectively (p < 0.01). The maximum isoflurane concentration ranged from 0.7 ppm to 2.8 ppm and from from 8.3 ppm to 43.7 ppm in group S and NS respectively.

**Conclusion:**

This double mask can be used to induce inhalation anesthesia in dogs. Scavenging from the mask significantly decreases the amount of waste anaesthetic gas concentrations in the breathing zone of the anesthetist. Therefore, such a system can be recommended whenever induction or maintenance of general anesthesia by mask is considered.

## Background

The induction of general anesthesia by administration of volatile anesthetic agents via a mask is an established procedure in many animals, also in dogs. Although it is possible to maintain inhalation anesthesia via the face mask it is generally advised for safety reasons to do this only for short procedures and to proceed to endotracheal intubation for maintenance instead. During application of a face mask it is almost inevitable that some anesthetic escapes at the interface with the head, even with a carefully executed technique. This causes contamination of the workplace with variable amounts of volatile agent. Inhalation of waste anesthetic gas concentrations is a concern not only for the health of the anesthetist who is working in close proximity to the animal's head but also for everyone else in the workplace.

There is evidence that exposure to trace amounts of volatile anesthetics may constitute a health hazard and be associated with neurobehavioral effects. Exposure to trace concentrations of isoflurane can cause damage to sister chromatids in an order of magnitude that is equivalent to the effect of smoking 20 cigarettes per day [1]. People exposed to waste anesthetic gas experience significantly longer reaction times compared to a control group without exposure [2]. A review of studies concerning health effects of volatile anesthetics [3] concludes that, based on the literature, it is impossible to state whether or not occupational exposure to volatile anesthetics constitutes a threat to health of operating room personnel. However to decrease the risk of potential health damage, the level of contamination at the workplace should be kept at a minimum level [4,5]. Nowadays, most countries have regulations in place and clearly specify the allowed maximal exposure levels to various agents by the personnel.

In human medicine Nilsson et al [6,7] proposed a close scavenging system to reduce workplace contamination. Reiz [8] introduced the double mask system in human medicine in 1986. This type of scavenging mask enables simultaneous delivery of anesthetic gas and scavenging of escaping waste gas.

In veterinary medicine, scavenging double mask systems are commercially available for use in very small animals like laboratory rodents but also eg small birds. A scavenging double mask featuring a special demand valve was proposed for piglet castration under inhalation anesthesia [9]. To the best of the author's knowledge, use of a scavenging double mask system has not been reported in dogs.

In the present study, a custom made scavenging double mask was used to induce inhalation anesthesia in dogs. The waste anesthetic gas concentrations in the breathing zone of the anesthetist were measured with and without active scavenging from the mask.

## Methods

### Animals

Twelve university-owned beagles enrolled in a dental research project and scheduled to undergo general anesthesia were included in the study. The project was discussed and approved by the University's ethical committee. The dogs were 2.7 ± 1.2 (mean ± SD) years old and weighed 14.2 ± 2.2 kg. After clinical examination, all the dogs were classified as ASA I patients. Food was withheld for 12 hours but the dogs had constant access to water.

### Anesthetic procedure and experimental design

All dogs were premedicated with 10 μg/kg medetomidine (Domitor^®^, 1 mg/ml, Pfizer, Orion Corporation, Espoo, Finland) and 0.2 mg/kg butorphanol (Butomidor^®^, 10 mg/ml, Richter Pharma AG., Wels, Austria) intravenously administered via a catheter in the right cephalic vein.

Five minutes later mask induction of anesthesia was initiated using isoflurane in 100% oxygen (Isoba; Essex Tierarznei, Munich, Germany). The gas mixture was delivered to the mask via a coaxial Mapleson D breathing system (Intersurgical GmbH, Sankt Augustin, Germany) and the flow was set at 150 ml/kg/min. The anesthetic machine (Ohmeda Excell 210 SE, Madison, Wi, USA) was equipped with a precision isoflurane vaporiser (Datex-Ohmeda Isotec 5, Madison, Wi, USA). Excess gas from the pop-off valve of the machine was directed to a dedicated charcoal cannister for adsorption of isoflurane (Cardiff Aldasorber, Bradwell, UK). All mask inductions were performed five minutes after premedication by the same anesthetist in the same induction room. Only one procedure was done and no other anesthetics were administered in the room. At all occasions the double mask was connected to a scavenging unit. In 6 dogs (group S) the scavenging mode was turned on and in 6 dogs (group NS) scavenging was disabled. The scavenging equipment produced some noise and small vibrations of the mask. Therefore the anesthetist performing the mask induction was inevitably aware of whether the scavenging mode was turned on or not.

The duration of induction time was standardised to 8 minutes. Thereafter the mask was removed and the trachea was intubated allowing maintenance of anesthesia in a surgery room. After termination of the procedure each dog was given 50 μg/kg atipamezole intramuscularly (Antisedan^®^, 5 mg/ml, Pfizer, Orion Corporation, Espoo, Finland) and was allowed to recover in a recovery room.

### The double mask and scavenging system

**Figure 1:**
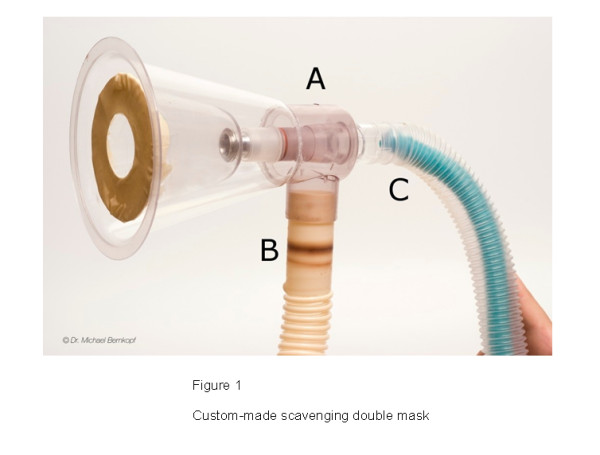
**Custom-made scavenging double mask featuring a latex diaphragm and a special connector (A) which connects the scavenging hose with the port for the outer mask (B) and the anesthetic system with the port for the inner mask (C)**.

The mask induction was performed with a custom-made double mask (Figure 1). This double mask was made by using the connection piece of a commercially available double mask from human medicine (Medicvent AB, Umeå, Sweden) and two clear plastic masks that are used in veterinary medicine (Midmark Corporation, Versailles, Ohio, USA).

The outer mask was permanently fixed to the connection part, whereas the inner mask could easily be disconnected in order to allow exchange with different sizes of inner mask. For the inner mask the provided rubber diaphragm was replaced by a latex diaphragm made from a surgical glove. The circumference of the dogs' noses was similar (20.3 ± 1.4 cm) and the same inner mask (outer diameter 23 cm) was used for all the mask inductions.

The connection piece of the mask has two ports. One port was connected to the breathing system and the second port was always connected to the scavenging system also when scavenging was not activated (Figure 1).

The scavenging system consisted of a commercially available fan unit (Medicvent AB, Umeå, Sweden) which in the present study directed the scavenged gas via flexible tubing to the open air. The scavenging flow rate selected was 27 m³/h. This rate is recommended by the manufacturer for humans when using small face masks or in the presence of low respiratory gas flows (Manual double mask system; Medicvent AB, Umeå, Sweden).

### Mask induction

Mask induction was performed with the dogs in right lateral recumbency. The inspired isoflurane concentration was increased by turning the dial of the vaporiser. This was performed in 1 volume% steps every 30 seconds starting with 1% until a 5% dial setting was reached. This setting was maintained until the lid reflex disappeared, and then changed to 2%. Eight minutes after the start of mask induction the mask was removed and the anesthetist performed the endotracheal intubation and reconnected the patient to the anesthetic circuit.

### Isoflurane measurement

The gas concentrations of isoflurane were measured with photoacoustic spectroscopic analysis of continuously sampled room air. The measuring device used was a Brüel and Kjær multigas monitor 1302 (Brüel and Kjær, Nærum, Denmark) calibrated according to the manufacturer's specifications prior to the study. The sampling line used was two meter long and made of polytetrafluorethylene.

Although the sampling mode of the analyzer was set "continuously" (30 cm^3^/sec), the internal processing for measurements enables measurements for the waste gas concentration to be effectively performed only approximately every minute. The measured concentration of isoflurane was expressed in parts per million (ppm). From the time of premedication until intubation, the proximal end of the probe was fixed on the shoulder of the anesthetist to measure the isoflurane concentration in the anesthetist's breathing zone.

### Data recording and analysis

The mean value of the isoflurane concentration in the anesthetist's breathing zone before mask induction (5 min) and throughout the entire period of mask induction (8 min) was calculated (mean ± SD).The maximal values during this period were recorded. The values between premedication and induction (five min) were defined as baseline values. Additionally the isoflurane concentration during intubation was recorded.

All data were transferred to a personal computer for post-hoc analysis using SPSS 16.0 for Windows. Data are expressed as mean ± SD and percentage of baseline. The normality of the distribution was evaluated by using the Kolmogorov-Smirnov test. Within group and between group comparison was done with student's t-test und the Mann-Whitney U test.; p < 0.05 was considered significant.

## Results

Measurement results are presented in table 1.The double mask was well tolerated by all dogs and anesthetic induction unremarkable. Except for data recorded at the time point of intubation, all data were normally distributed. The baseline concentrations were not significantly different between the groups. During mask induction the mean and the maximum isoflurane concentrations in group NS were significantly higher than in group S (p < 0.01). During intubation at the moment the mask was not in use, the waste gas concentrations were also significantly increased compared to baseline (p < 0.01) but not different between the groups.

**Table 1:** Isoflurane concentrations in the anesthetist's breathing zone

Group S				Group NS			
**Dog**	**Baseline**	**Induction mean**	**Induction max**	**Dog**	**Baseline**	**Induction mean**	**Induction max**

1	0.5 ± 0.1	0.7 ± 0.1 (122)	1.0 (180)	7	2.9 ± 2.0	16.9 ± 14.3 (587)	39.4 (1365)
2	1.2 ± 0.1	1.5 ± 0.5 (127)	2.2 (191)	8	1.5 ± 0.1	10.4 ± 7.6 (692)	23.7 (1587)
3	1.7 ± 0.4	2.1 ± 0.3 (128)	2.7 (162)	6	2.3 ± 0.2	19.4 ± 16.1 (858)	43.7 (1936)
4	2.5 ± 0.4	2.6 ± 0.5 (107)	3.2 (130)	10	3.1 ± 0.7	8.8 ± 4.0 (289)	15.3 (503)
5	2.5 ± 0.2	2.8 ± 0.6 (115)	4.1 (165)	11	1.9 ± 0.1	8.5 ± 4.8 (461)	17.1 (925)
6	2.6 ± 0.2	2.5 ± 0.3 (96)	2.8 (108)	12	2.3 ± 0.3	3.2 ± 2.1 (141)	8.3 (366)

	mean ± SD 1.8 ± 0.8	mean ± SD 2.0 ± 0.8	mean ± SD 2.7 ± 1.0		mean ± SD 2.3 ± 0.6	mean ± SD 11.2 ± 6.0^a,b^	mean ± SD 24.6 ± 14.1^a,b^

Mean ± SD isoflurane values for Group S (scavenging activated) and Group NS (scavenging disabled) in parts per million (ppm) and percent of baseline value. Mean isoflurane concentration during five min before (baseline) and eight min of mask induction (Induction mean).Maximum isoflurane concentration recorded during mask induction (Induction max).

^a^: significantly different from group S (p < 0.01)

^b^: significantly different from the baseline (p < 0.01)

## Discussion

The results of this study demonstrate that the novel double mask for dogs can be satisfactorily used for mask induction and that simultaneous scavenging significantly reduces workplace contamination. This is in line with reports from human anesthesia where the anesthetist's exposure was reduced from 2.9 ± 1.1 to 0.5 ± 0.1 ppm and from 134 -764 ppm to 9-42 ppm for halothane [8] and nitrous oxide respectively [10].

The double mask presented in this study was easily applied and maintained in place in all cases and allowed normal induction of anesthesia. Furthermore it also allowed simple simultaneous scavenging which was efficient in keeping workplace contamination low.

The inner mask of the double mask system should assure an airtight seal with the contours of the dog's muzzle. Some veterinary masks come without a diaphragm and rely heavily on a degree of malleability in order to establish a sealed connection between the mask and muzzle [11]. The waste gas contamination during mask induction in rats was significantly reduced when the mask was equipped with a latex diaphragm [12]. For the double mask in the present study the rigid rubber diaphragm of the inner mask was replaced by a latex diaphragm made from a surgical glove. This was considered more comfortable for the dogs than the rigid rubber when firm application is necessary to realise a tight seal.

There exist recommendations concerning the acceptable maximum exposure to waste gas concentrations of anaesthetic, which differ in different countries. Time weighted average (TWA) values are proposed over an 8 h period and over a 15 min period (Short-Term Exposure Limit, STEL). The STEL should not be exceeded at any time during a workday even if the 8 hour TWA is within the threshold limit value. The STEL values for isoflurane as a sole anaesthetic agent in European countries varies from 4 to 20 ppm whereas the 8h TWA varies from 2 to 50 ppm [5,13]. In general no advice is given for instantaneous maximum values. In the study presented the mean isoflurane concentration over eight minutes did not exceed 10 ppm in the presence of scavenging and remained under 20 ppm without scavenging. However, the values represent calculated means of values recorded over eight minutes and cannot be compared directly with officially recommended STEL thresholds who represent time weighted averages over 15 min. These relatively low values suggest that under the conditions of the study in general an efficient seal was realised with the latex diaphragm. Nevertheless peak values > 20 ppm have been recorded in 3 out of 6 anesthesia inductions without active scavenging and none with active scavenging.

This study has some limitations. The automatic measurement of anaesthetic gas concentrations was performed only every minute and in presence of rapidly changing concentrations. Therefore, the measured "maximal" values may in fact not represent the real peaks and may underestimate effective pollution.

The study was not randomised and could not be blinded. Given the similar baseline values of both groups it is unlikely that randomisation would have altered the results of the study. The anesthetist was aware of the potential health hazard linked with mask induction and was instructed to aim for the best possible seal of the mask in every dog. The degree of leakage at the level of a mask can also be influenced by the gas flow used. Low flows are associated with less occupational exposure [14]. However, during the induction of inhalation anesthesia, relatively high flows are recommended in order to quickly achieve the desired inspired concentrations of oxygen and volatile agent. In the present study, the flow rate was fixed at 150 ml/kg/min as recommended for Mapleson D systems [15]. This represents a total fresh gas flow for the dogs in the study of 1.7 L to 2.7 L/min. It is possible that if higher gas flows had been used like eg 3 L/min recommended for mask induction [16] the degree of workplace contamination would have been higher.

The dogs were markedly sedated and tolerated the application of the mask in lateral recumbency without resistance. However compliance to mask induction might be less in conscious or less sedated patients and the necessary tight connection between the mask and muzzle more difficult to achieve. In such cases, workplace contamination is likely to be higher than what was found in the present study. Also the mask was particularly suited for mesocephalic dogs allowing a good seal and reduced dead space. In brachycephalic dogs the fitting of the mask may be less optimal and this requires further study.

There was no simultaneous measurement of waste gas at the table level or at other locations of the induction room. This would be of interest to evaluate the exposure of other people working in the induction room.

It was also noted that during intubation the waste gas concentration in the breathing zone still exceeded baseline level although the mask and anesthetic system were removed. This was likely caused by the presence of isoflurane in the expired breaths of the dogs the face of the anesthetist being directly in front of the dog's mouth during this procedure. This fact jeopardises in terms of pollution the efforts to keep contamination low during mask induction. There seems to be no way to avoid this contribution to exposure but it is suggested that during intubation an assistant can hold the double mask in the scavenging mode as a scavenging hood close to the dog's mouth.

## Conclusion

The scavenging double mask presented in this study can be used in dogs and significantly decreases the waste anesthetic gas concentrations in the breathing zone of the anesthetist. Therefore, such a system can be recommended whenever mask induction or the maintenance of general anesthesia via a mask is considered in dogs.

## Competing interests

The authors declare that they have no competing interests.

## Authors' contributions

YM planned the application of the double mask-concept for mask induction in dogs and organised basic equipment. HS assembled and tested the first prototype with advice from PC. SF set up, collected and analysed the data of the study with advice from PC and prepared the major part of the manuscript. YM performed critical review and finalisation of the manuscript. All authors read and approved the final manuscript.
